# How to … use qualitative research to change practice

**DOI:** 10.1111/tct.13085

**Published:** 2019-09-05

**Authors:** Anu Kajamaa, Anne de la Croix, Karen Mattick

**Affiliations:** ^1^ Faculty of Educational Sciences University of Helsinki Finland; ^2^ LEARN! Academy Vrije Universiteit Amsterdam the Netherlands; ^3^ Amsterdam UMC VUmc School of Medical Sciences Research in Education the Netherlands; ^4^ Centre for Research in Professional Learning University of Exeter UK

## Abstract

The ‘How to …’ series focuses on how to do qualitative research. But how can qualitative research enhance patient care? This paper aims to support health care practitioners, educators and researchers who are interested in bridging the gap between research and practice (both clinical and educational), to guide improvements that can ultimately benefit patients. We present action research and The Change Laboratory method as two approaches that typically involve qualitative research and have potential to change practice, blending scientific inquiry with social action. These approaches establish close research–practice partnerships and help answer tricky ‘why’ and ‘how’ questions that may unlock deep insights to enhance learning and patient care.

… how can qualitative research enhance patient care?

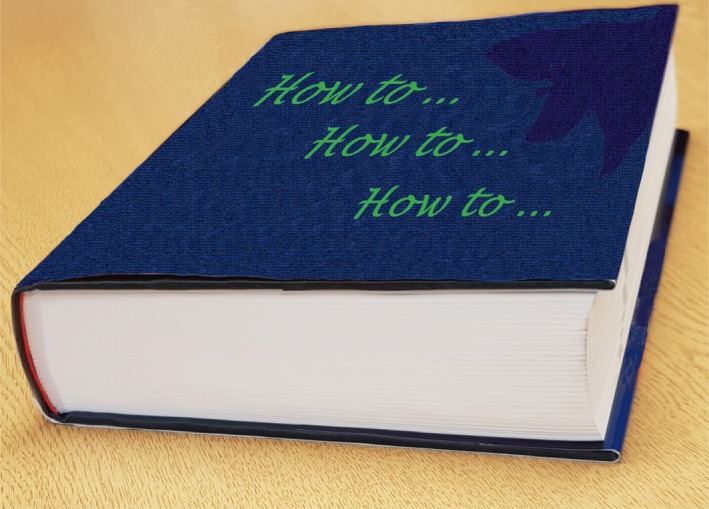

## Introduction

As health care researchers, educators or practitioners, we may become frustrated by the gap between research and practice (both clinical and educational). To help facilitate practice change, qualitative research can be co‐designed and co‐delivered in partnership with research participants, in this case health care practitioners. The gap between research and health care practice can be reduced by the immersion of the researcher in the practice environment, for example, through researchers’ observations of clinical practice and the learning environment, including conversations and interactions, in its local context. The research questions may be formulated collaboratively with the research partners from the practice site.^1^ In the approaches to qualitative research described in this paper, the researcher aims to give voice to the participants in order to provide insights into each other's thoughts and beliefs. For this purpose, she or he may hold meetings and conversations, which can help different parties to make sense of their experiences of their daily encounters with complex processes and practices, their teams, their institutional vision and so forth. Moreover, the researcher does not seek to implement predefined change mandates but supports the participants to establish a joint dialogue,^2^ and to discuss issues such as their existing norms, division of labour and roles. The ‘why’ and ‘how’ questions proposed by the researcher can aid participants in their thought process and articulation of problems they encounter.

The gap between research and health care practice can be reduced by the immersion of the researcher in the practice environment …

Despite the increasing popularity of such participatory methods, and despite the involvement of several stakeholders, it is still frequently difficult to bring about change in health care practices for improved patient care. The gap between research and practice may be detected at multiple levels. According to existing studies, health care often involves opposing demands, such as cost‐effective delivery in tandem with high‐quality care.^3^, ^4^ This leads health care managers to introduce change initiatives and evaluate their effects, comparing them to the original situation and the intended outcomes. These efforts are typically based on quantitative measuring systems and management models that emphasise rationalisation, marketisation and cost‐effectiveness^5^ and are not always effective^6^ because top‐down change efforts can easily fail to accommodate the needs of the practitioners and the complexity of health care organisations.

Intervention techniques that draw on qualitative data and participatory methodologies, and that may provide a nuanced understanding of problems in local contexts, are thus increasingly needed.^7^ This involves paying attention to issues such as the professional boundaries between the different health professionals involved in care processes, which may have formed over a long time, since they may influence the process of delivering care. Increased attention also needs to be directed to the work and learning of health care students and trainees who often experience challenges within these complex environments.^8^ Acknowledging the possibility of unexpected consequences when attempting to change complex processes and practices also becomes important. Below, we present two approaches that provide ways for researchers and practitioners to collaborate flexibly and effectively when addressing complex workplace problems and needs for change.

## Two Uays of Doing Practice‐Based Research

Action research and The Change Laboratory method are applied in the study of work processes and practices without seeking to implement top‐down directed change mandates, and are thus linked to the democratisation of working life. They focus on qualitative transformation of practice within entire organisational systems, include multiple cycles of change, and involve participants with diverse and often under‐represented views. Aiming to facilitate practice improvement, they study local development needs and problems, such as communication challenges, bottlenecks in care practices, such as a delay in moving a patient from a ward to the operating room, and other problems, such as absence of staff due to sick leaves and lack of substitutes. They focus on social interaction, social practices, social changes and new forms of collaboration to generate new knowledge and practices. These are typically impactful since they are highly relevant to the research context and can promote high‐quality care.^1^, ^9^

Action research projects and The Change Laboratory method allow multiple viewpoints to be presented that can help build a shared memory of events and processes, which is essential to the identity and integrity of a workplace community. Both action research and The Change Laboratory method embrace a tension between local solutions and transferable knowledge and aim to make the results of research more generally applicable.^1^, ^10^, ^11^, ^12^ Both approaches also call for deep cognitive and emotional engagement from the researcher, for example, in facilitating interventions in near crisis contexts.

### Action research

Action research originates from the work of German‐American psychologist Kurt Lewin (1947) and has been defined as ‘an iterative process in which researchers and practitioners act together in the context of an identified problem to discover and effect positive change within a mutually acceptable ethical framework’.^1^ The focus is usually on societal practices and their improvement, and work is always undertaken in collaboration with the participants. Theorising within action research is understood as a form of practice, with theory and practice seen as integrated and as a generative transformational cycle that has the potential for infinite self‐renewal.^13^ Action research has been used widely within health care^14^ and medical education research.^15^ For an example, see Box 1.

Box 1 **Examples of projects**

*An action research project*

Lingard et al. improved interprofessional care by changing a model of service delivery.^1^ The project led to the introduction of an interprofessional care coordinator in an acute medical inpatient setting. Data were collected via participatory observations, field notes, semi‐structured interviews, focus groups with key stakeholders and key documents from the research site. This action research process consisted of three main cycles, in which the researcher and participants jointly reflected on the research questions, analysis and findings, and defined the core ideas for practice development. Their joint work included topics such as defining the role of interprofessional care coordinator, issues of accountability and how to enhance interprofessional working.

*The Change Laboratory project*

In a surgery and intensive care unit in Finland with high sickness absence rates and other problems paralysing activity, the participants gave ‘social sense’ to the near crisis situation. The researchers utilised interviews, observations, documents and other data to deliberately foster learning. They created a ‘mirror’ depicting the contradictions in the daily activity of the participants, hampering high‐quality care, and in the intervention triggered the collective reflection of the work activity from the patient's perspective. The intervention followed a learning cycle, including actions of questioning, criticising and rejecting some aspects of the accepted practice and existing wisdom. The collective analysis of the problem evoked ‘why?’ and ‘how’ questions, which enabled a new explicit, simplified model to be constructed that offered a framework for practice change. The model was tested to understand its potentials and limitations, before a period of reflection and evaluation allowed the model to be consolidated into a new stable form of practice. The unit overcame the crisis, significantly increased its efficiency and strengthened its community.^10^

Action research projects and The Change Laboratory method allow multiple viewpoints to be presented that can help build a shared memory of events and processes …

### The Change Laboratory

Another way to facilitate practice change is the research‐assisted method called The Change Laboratory, which stems from activity theory, which has been used extensively to study workplace learning^11^ and increasingly also in medical education.^8^ Since the late 1990s, The Change Laboratory has been utilised successfully in health care research and development,^10^, ^16^ to explain the current situation and the need for change. This approach harnesses the ‘disturbances’ and ‘contradictions’ – in other words, things in the workplace that aren't happening as intended – in order to understand what is hampering the activity. Through this process, new collective insights can develop, leading to practice change. This method supports the development of models enhancing collective learning, in other words, learning of ‘something that is not yet there’.^10^, ^11^, ^12^ Activity theory can also be used in the analysis of action research projects.^9^, ^17^ For an example of a Change Laboratory, see Box 1.

## Benefits and Challenges of Using Action Research and the Change Laboratory

**Figure 1:**
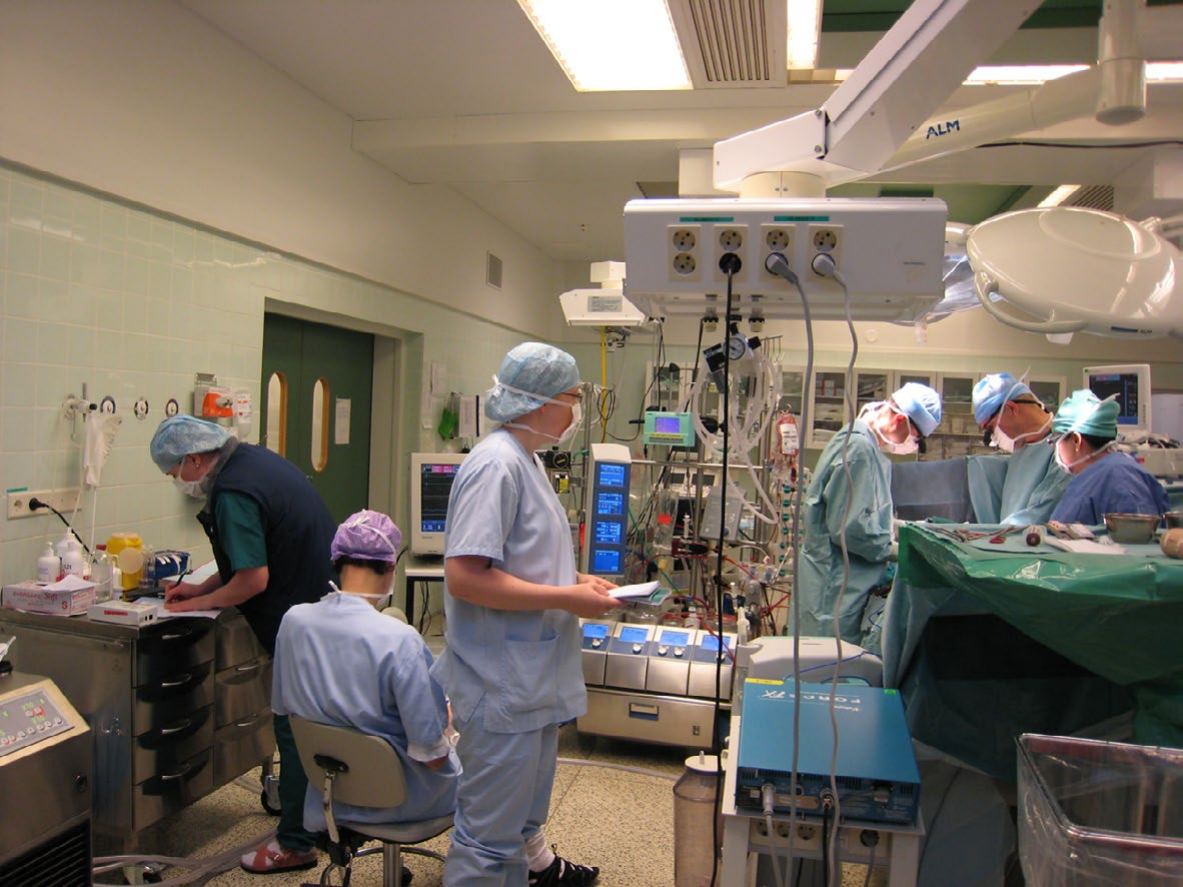
A researcher conducting ‘ethnography of change’ by observing practices and writing field notes prior to The Change Laboratory intervention in a surgical unit.

There are many strategies and tools that researchers can use when doing the type of work we describe here. Many of the required qualities might already be in your skill‐set, as they draw on curiosity, interest, listening and building trust. Should you want to start your own action research or The Change Laboratory, see Box 2 and Figure 1 for some advice to support novice researchers.

Box 2 **Advice to support novice researchers wishing to undertake practice‐based research**

- Find a mentor or team to collaborate with and to support you in undertaking the work
- Invest time in reading some key references about your chosen methodological approach and seek advice from experts
- Spend time in the field to observe, deeply involve, educate and empower research participants throughout the project, from identifying the problem to disseminating results
- Build trust with participants and consider ethical issues critically, such as role of the researcher, anonymity of participants, and ‘ownership’ of findings
- Collect various types of data, through interviews, meetings and observations. Relevant documents may also be gathered and analysed
- Write field notes during the visits, which can function as an important stimulus for self‐reflection
- Keep data safely – these may take the form of photographs, audiorecordings or video‐recordings, quantitative data such as surveys or workplace data
- Involve research participants. Both in action research and The Change Laboratory, the research participants may also be involved in data collection, for example, in undertaking interviews
- Collect disturbances. Keep diaries of disturbances encountered in everyday practice, which may be used as ‘mirror material’ in The Change Laboratory, helping participants to ‘look again’ at everyday issues that cause struggles and to redesign their collective activity. This way of collecting data has been defined as ‘ethnography of change’ (see Figure 1)
- Listen to stories! The data analysis process often involves narrative methods, which depict ‘stories from practice’ and enable actions and interconnections within organisations to be explored

Action research and The Change Laboratory method offer both methodological opportunities and challenges. The Change Laboratory can be used to analyse data collected through action research, together with the research participants.^9^ It also provides a new way to look at the practice of health care in which contradictions offer the opportunity to examine challenges within and between different professional groups. Importantly, action research and The Change Laboratory both facilitate a process without predetermined results, and aim to aid the participants’ collective learning and sense‐making of the changing contextual demands. Thus, the outcomes are designed by participants as they work out expansive solutions to the problems within their workplaces.^10^ However, the complexity of care processes and the uncertainty about outcomes^8^ can create frustration, ambiguities and further tensions amongst participants, which can also be challenging for the researcher to manage. In addition, both approaches described here investigate situated cases, and people experiencing particular critical situations, so the findings might not be directly transferrable to other social or organisational settings.^1^ However, on balance, we believe the challenge of transferability and uncertainty of outcomes are worthwhile given the potential rewards.

Many of the required qualities might already be in your skill‐set, as they draw on curiosity, interest, listening, and building trust

## Conclusions

A good qualitative research project is original, rigorous and relevant^18^ – and it is the relevance for practice change that is the particular strength of the approaches described here. Action research and The Change Laboratory provide ways of ‘looking again’ at everyday issues that may cause struggles in delivering health care and health care education‐related practices. They help to answer tricky ‘why’ and ‘how’ questions in complex organisations and encourage participation and community development. Problems and tensions in work practices are seen as important ‘drivers’, providing a useful stimulus for change and refinement of clinical work.
